# A Case of Complicated Silicosis with a Complex Clinical Course in a Glass Manufacturing Worker

**DOI:** 10.1186/2052-4374-26-10

**Published:** 2014-05-15

**Authors:** Hee-Seok Yang, Jung-Il Kim, Byeong-Jin Ye, Tae-Jun Yoo, Sun-Woo Lee, Kap-Yeol Jung

**Affiliations:** 1 Department of Occupational & Environmental Medicine, College of Medicine, Dong-A University, Busan, Korea; 2 Department of Occupational & Environmental Medicine, College of Medicine, Koshin University, Busan, Korea

**Keywords:** Silicosis, Glass, Supraclavicular lymph node

## Abstract

We reported a case of complicated silicosis that occurred in a glass manufacturing plant worker who had presumably been exposed to low-concentration free silica for almost 20 years. To the best of our knowledge this report is the first in the Republic of Korea. The physician’s first impression was cancer since the enlargement of neck and supraclavicuar lymph nodes had clearly progressed and metastasis was suspected in ultrasonography. However, it turned out to be reactive hyperplasia and anthracosis. Although lung cancer was suspected and tests were performed in 2 hospitals due to repetitive cough and dyspnea, along with weight loss of approximately 10% over the course of 7 months, the patient was eventually diagnosed with complicated silicosis and pneumothorax occurred after 1 year. Herein, we report this case with a literature review.

## Background

Silicosis is a parenchymal lung disease that results from the inhalation of silicon dioxide, or silica, in crystalline form. Silica is a major component of rock and sand. Workers with potential for exposure are miners, sandblasters, foundry workers, tunnel drillers, quarry workers, stone carvers, ceramic workers, and silica flour production workers [1]. The industries in the Republic of Korea (hereafter, “Korea”) in which pneumoconiosis has traditionally occurred have been mainly metal and coal mining. Although the proportion of pneumoconiosis cases in the manufacturing sector has been increasing, the 2012 occupational accident statistics showed that 333 cases (92.8%) of pneumoconiosis occurred in the mining sector while only 24 (7.2%) did in the manufacturing sector, indicating that the mining sector still accounted for the vast majority [2].

Glass manufacturing processes generally consist of the blending process, in which the raw materials of glass are mixed; the melting process, in which the raw materials are melted at high temperatures; the forming process, in which the products are shaped, and the finishing process, in which the products are printed and finished. Among these processes, although blasting during the mixing and finishing processes may put workers at a high risk of exposure to free silica, it has been reported that the exposure concentration of crystalline free silica is low in glass manufacturing [3]. Therefore, although silicosis may also occur in glass manufacturing workers, few cases have been reported in other countries and none in Korea. Accordingly, since we identified a case in which a worker who had been working in the mixing process of glass manufacturing for 20 years showed complicated silicosis displaying various clinical characteristics such as pneumonitis and pneumothorax along with a suspicious finding of cancer metastasis in the cervical and supraclavicular lymph nodes, we report herein the case with a literature review.

## Case presentation

### The case

**Patient**: Male, 57 years old.

Hospital “A”

**Chief complaint**: Continuous cough for 1 month

**History of the present illness**: Pneumonitis (for which differential diagnosis of pulmonary tuberculosis was needed) was diagnosed by plain chest radiography carried out in a nearby hospital due to continuous cough for 1 month. The patient visited the Division of Pulmonology at the “A” University Hospital on the same day and was hospitalized.

**Past medical history**: No special findings.

**Personal history and family disease**: The patient had been smoking a pack of cigarettes daily for 40 years, and did not drink alcohol. There was no notable family disease.

**Physical examination**: Enlarged cervical lymph nodes were found bilaterally.

**Blood test**: Hemoglobin (Hb) was measured to be 12.6 g/dl in the complete blood cell count (CBC), which was a slightly decreased concentration, while the total proteins and albumin were in the normal range. The erythrocyte sedimentation rate (ESR) increased to 73 mm/h.

**Tumor marker**: Carcinoembryonic antigen (CEA) was 0.66 ng/ml, which was at the normal level, while neuron-specific enolase (NSE) increased to 19.31 ng/ml.

**Figure 1:**
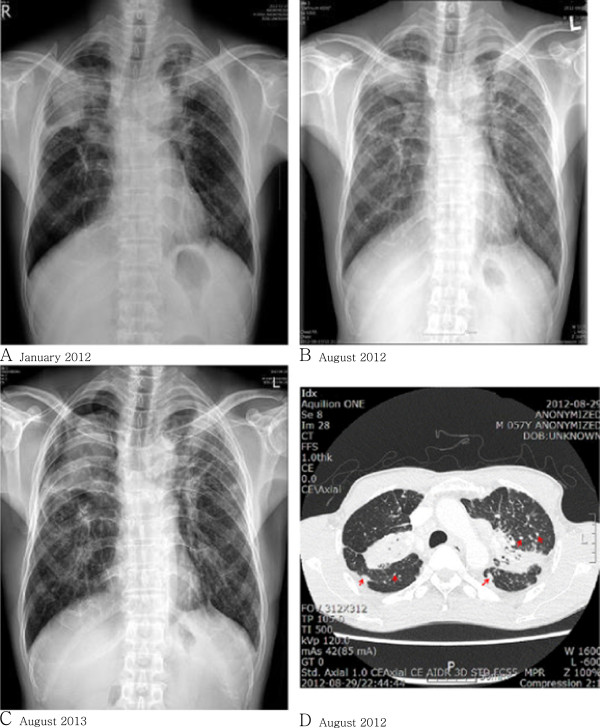
**Chest X-ray. A**. Pneumonitis or pulmonary tuberculosis in both upper lobes. A lung mass lesion in the right upper lobe was ruled out. **B**. Pneumoconiosis such as silicosis with progressive massive fibrosis (PMF). Slightly decreased densities around PMF were present since January 2012. A differential diagnosis for lung cancer, which is rarely considered, was performed. **C**. Pneumothorax, right. Underlying complicated pneumoconiosis, progressive massive fibrosis. HRCT. **D**. PMF, subpleural, and centrilobular silicotic nodules (arrows) are seen at both lung and pseudoplaque formation (arrow at pleural area) in the right lung.

**Chest images**: In the plain chest x-ray, potential pneumonitis or pulmonary tuberculosis was found bilaterally in the upper lobes of the lungs (discernment of a lung tumor on the right upper lobe was required) (Figure 1-A). Pneumonitis was most suspicious bilaterally on the upper part of the lungs from the chest computed tomography (CT). Based on the imaging study, we concluded that complicated silicosis was accompanied by pneumonitis. Sarcoidosis or pulmonary tuberculosis had to be considered if this pneumonitis was to be classified as a tumor.

**Figure 2:**
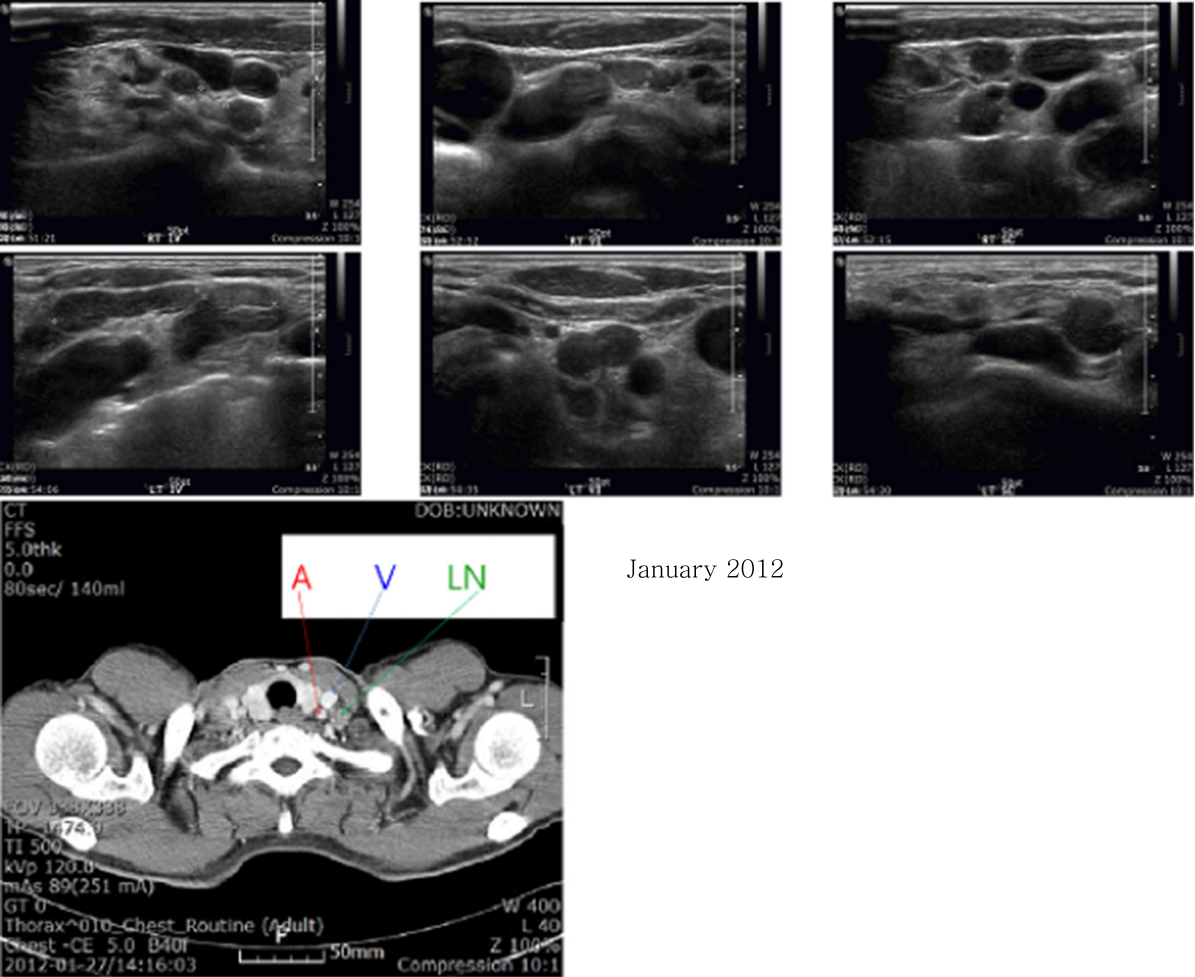
**Thyroid and neck sonography.** Enlargement of multiple lymph nodes appears like metastases at both neck levels IV and VI and the supraclavicular level. CT with enhancement. Lymph node enlargement about 2 cm in size at the left supraclavicular area. Abbreviation (arrows): A, common carotid artery. V, jugular vein. LN, supraclavicular lymph node.

**Findings from thyroid and cervical ultrasound**: There were no abnormal findings in the thyroid. The cervical level IV and VI lymph nodes and supraclavicular lymph nodes on both sides were enlarged, which raised a suspicion of cancer metastasis (Figure 2).

**Findings in excision biopsy of cervical lymph nodes**: Excision biopsy of the enlarged lymph nodes showed reactive hyperplasia and anthracosis.

**Bronchoscopic findings**: No endobronchial lesions were observed. The bronchial washing test gave negative results for acid-fast bacillus (AFB), AFB polymerase chain reaction (PCR), AFB culture test, and microscopic cytology. In the endobronchial ultrasound-guided transbronchial needle aspiration (EBUS-TBNA), atypical cells were observed on the right bronchial tubes. Immunohistochemistry (IHC) on the reactive lymphoid tissue of 2 lymph nodes produced negative results for CD 56, chromogranin A, and synaptophysin.

**CT-guided percutaneous needle biopsy (PCNB)**: In a biopsy of the lungs, malignant neoplasm was not found while fibrosis and anthracosilicosis were observed.

**Pulmonary function test**: The forced vital capacity (FVC) was 67%, and forced expiratory volume in one second (FEV_1_) 59%, and FEV_1_/FVC (%) 64%, indicating a mixed ventilatory defect.

**Diagnosis and progress**: Lung cancer was suspected, accompanied by pneumonitis. As the dyspnea symptoms had improved somewhat, the patient was discharged and positron emission tomography - computed tomography (PET-CT) was planned as an outpatient follow-up examination. However, the patient has not returned to Hospital “A” since then.

Hospital “B” (7 months later)

**Chief complaints**: cough, dyspnea (repeated worsening and improving for 7 months)

**History of the present illness**: The patient had been discharged from Hospital “A” voluntarily without a definitive diagnosis during the medical evaluation he was undergoing due to continuing symptoms of dyspnea, cough, and fatigue during exercise that had started 7–8 months before. Then he visited the Department of Pulmonology of Hospital “B” in August 2012 with repeated worsening of symptoms.

**Physical examination**: At the time of admission, his body temperature was 36.5°C, pulse 98 beats/min, blood pressure 105/60 mmHg, respiration rate 20 breaths/min, and with decreased bilateral respiratory sounds in the lungs. While his nutritional status was good and mental status was normal, he looked chronically ill and his whole body was weak. He had difficulty in breathing even when sitting still, and the symptoms worsened when he lay on the left side. He had lost 5 kg—from 55 kg to 50 kg—since January 2012.

**Chest images**: Small opacities were observed throughout the whole lung in plain chest radiographic images, and a large opacity was observed in the right upper lung, interpreted to be pneumoconiosis (ILO classification u/u, 2/3, A) (Figure 1-B). Pneumonic infiltration was observed around the large opacity. Pneumoconiosis (silicosis pattern) accompanied by progressive massive fibrosis, along with reactive lymph nodes, were found in the chest CT, which, in addition, required the discernment of lung cancer. Nodules characteristic of silicosis were observed in the centrilobular and subpleural parts of the lung in the chest high resolution CT (HRCT) (Figure 1-D).

**Pulmonary function test**: The forced vital capacity (FVC) was 57%, and forced expiratory volume in one second (FEV_1_) 47%, and FEV_1_/FVC (%) 66%, indicating a mixed ventilatory defect. The carbon monoxide diffusing capacity (D_L,CO_) was decreased to 61%.

**Findings from electrocardiogram and echocardiography**: The ejection fraction (EF) was normal at 60-64%, and there were no specific findings regarding cardiomobility.

**Blood test**: HB was slightly below normal at 11.9 g/dl, and the erythrocyte sedimentation rate (ESR) was 120 mm/h and c-reactive protein (CRP) 4.31 mg/dL.

**Figure 3:**
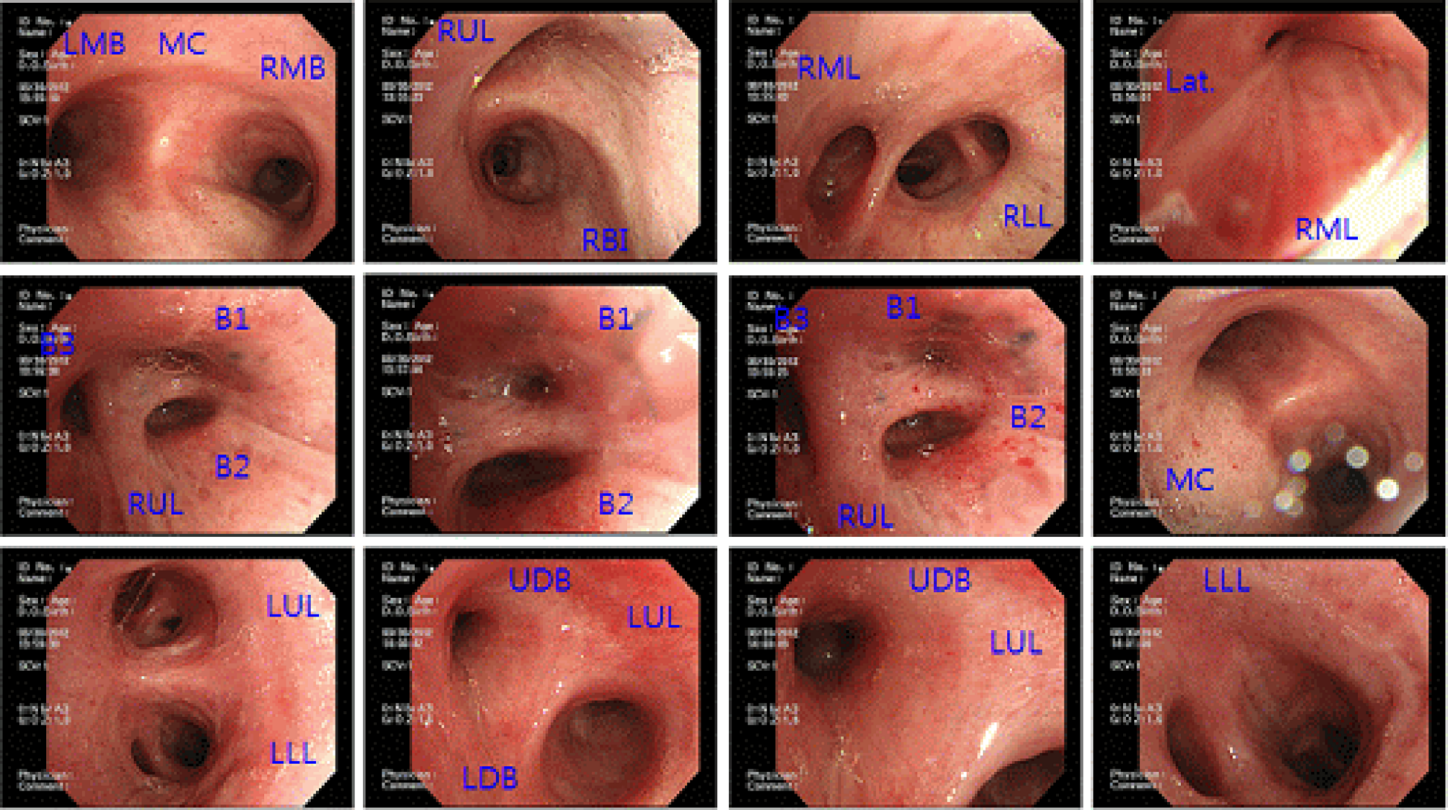
**Bronchoscopy.** Fibrosing stenosis at the apical, posterior, anterior segmental branch of the right upper lobe, lateral segmental branch of the right middle lobe, and apicoposterior segmental branch of the left upper lobe. Abbreviations. MC: main carina, LMB: left main bronchus, RMB: right main bronchus, LUL: left upper lobe, LLL: left lower lobe, RUL: right upper lobe, RML: right middle lobe, RLL: right lower lobe, RBI: right bronchus intermedius, Lat: lateral segmental branch, B1: apical, B2: posterior, B3 anterior, UDB: upper division bronchus (apicoposterior + anterior), LDB: lower division bronchus (lingular).

**Bronchoscopic findings**: The segmental bronchi within the right middle, right upper, and left upper lobes had become narrower (Figure 3), and a malignant cell test, AFB smear and culture tests, and AFB PCR test in broncho-alveolar lavage fluid were performed, all of which were negative.

**Job history and work environment (based on personal statement)**: The patient was referred to the Division of Occupational and Environmental Medicine and applied for occupational accident coverage since silicosis was suspected after excluding lung cancer. The patient's job history showed that he had been working in rice farming and greenhouse strawberry farming until 1992, and at the time, there had been no facilities that could expose him to dust, such as a cement factory or mine near the residence or farmland. For approximately 20 years since then, he had been engaged in the task of blending raw materials for manufacturing glass containers, and he stopped working in glass manufacturing after being admitted to Hospital “A”.

His main task was to put silicon dioxide, the major material comprising glass containers, along with additives including soda and calcium carbonate, into an automatic mixer, and to operate the machine 1–2 times a day for about 10 minutes. He was also engaged in other tasks such as product packaging and transport in addition to blending work, while neither grinding nor cutting glass was included in the manufacturing process.

The blender was placed indoors and the ceiling of the workshop was partially open. The workers used a dust mask onto which a handkerchief was attached during work.

**Diagnosis and progress**: The patient had been diagnosed with silicosis, and was discharged since the dyspnea symptoms had improved. He received outpatient treatment at the Division of Pulmonology and underwent a consultation on the occupational accident in the Department of Occupational and Environmental Medicine.

Hospital “B” (12 months later)

**Chief Complaint**: Acute dyspnea had occurred a week earlier.

**History of the present illness**: The patient presented to the emergency room because acute dyspnea had occurred while having suffered a severe cough due to a cold for 4—5 days.

**Past medical history**: The patient had been undergoing regular examinations due to pneumoconiosis (silicosis) accompanied by progressive massive fibrosis.

**Physical examinations**: When the patient came to the emergency room, he was clearly conscious with the temperature of 36.2°C, a pulse rate of 102 beats/min, blood pressure of 110/70 mmHg, a respiration rate of 20 breathes/min, 97% oxygen saturation of arterial blood. The height was 164 cm and the weight 48 kg, which had been reduced by 2 kg since August 2012.

**Arterial blood gas analysis (emergency room)**: The analysis showed that the pH was 7.419, pO_2_ 69.8 mmHg, pCO_2_ 32.7 mmHg, bicarbonate 20.7 mEq/L, O_2_ saturation 93.3%, and base excess −3.0 mEq/L.

**Chest Images**: In the chest X-ray images, pneumothorax on the right side and pneumoconiosis were observed (Figure 1-C). In the chest CT, pneumothorax on the right side, localized pulmonary edema in various sites, complicated pneumoconiosis accompanied by progressive massive fibrosis, and mediastinal reactive lymph nodes were observed.

**Examination findings**: An AFB smear examination of sputum, AFB culture test, and sputum culture test all gave negative results.

**Pulmonary function test (after treating pneumothorax):** The FEV_1_/FVC (%) was 63.8%, FVC 75.0%, and FEV_1_ 59.1%, indicating mixed ventilatory defects

**Clinical progress**: Pneumothorax and other symptoms have been improved by the oxygen therapy, and thus the patient was discharged after the 7 days of hospitalization. Follow-up examinations ensued, and the patient applied for occupational accident rehabilitation coverage.

## Conclusion

Although glass manufacturing has been known to cause silicosis by generating crystalline free silica, actual reports of the occurrence in Korea could not be found in any search. In addition, there have been only few reports suggesting that the elevation of the tumor marker NSE was involved in cases in which cervical and supraclavicular lymph node metastasis was suspected in silicosis patients. Burgess (1995) [4] reported that silicosis happens very rarely since sand is used in glass plants as a raw material. The glass manufacturing sector mostly consists of small-scale businesses, and 25,723 employees worked at 1,501 plants in 2005 in Korea [5].

The average exposure concentration of crystalline free silica is predicted to be 0.011—0.017 mg/m [3] in glass manufacturing, which is less than a third of the permissible exposure limit (Threshold Limit Value - Time Weighted Average, TLV-TWA; 8 h) by the Ministry of Employment and Labor [3,6], and thus it can be concluded that this sector does not have a high exposure concentration. Latency and the progression pattern of silicosis vary according to concentration of inhaling dust and the accumulated exposure amount of dust [7]. In the case of this report, it is likely that the worker was mainly exposed to crystalline free silica during the process of putting the main ingredient of glass containers, silicon dioxide, and additives, soda and calcium carbonate, into an automatic mixer. In addition, it appeared that the patient had worked in a poorly ventilated work environment for 20 years without properly wearing a dust mask, all leading to silicosis.

Crystalline silica was classified within Group I by the International Agency for Research on Cancer (IARC) [8], and newly included in the expanded list of carcinogenic substances of the revised enforcement ordinance of the Industrial Accident Compensation Insurance Act in June 2013 in Korea [9]. Progressive massive fibrosis is typically found in the upper part of lungs, showing “angel wings” patterns, and often mistaken for lung cancer [10]. It has been reported that if patients with silicosis smoke, the relative risk of lung cancer was 4.47 times higher [11]. There are approximately 600 lymph nodes in the human body, and 1% of all cases of lymphadenopathy are supraclavicular lymphadenopathy [12]. It has been reported that metastatic cancer or cancer accounted for 64-72% and reactive lesions for 10 ~ 19% if supraclavicular lymphadenopathy was observed. In case that cancer developed from supraclavicular lymphadenopathy, the origins of cancer have been reported to be the lung (22%), breast (16.4%), neck (11%), esophagus (8.6%) and unknown origin (13.3%) [13,14]. In the case of this report, lymph glands were felt on bilateral cervical parts in the physical examination along with such findings in the images, and bilateral cervical and supraclavicular metastatic lymph nodes were suspected in the ultrasonography. Moreover, a slight increase in NSE level was observed in the blood test. All of these lead to the suspicion of cancer. The lung biopsy is needed when clinical diagnosis is difficult, and an open-lung biopsy has been sometimes preferred due to the possibility of the occurrence of pneumothorax after the lung biopsy using bronchoscopy [15]. However, cancer was not confirmed in cervical lymph node biopsy, bronchoscopy and the lung biopsy, and the patient was discharged after the treatment of pneumonitis. This case is an example showing that it is difficult to distinguish silicosis from cancer, if the patient with complicated silicosis displays metastatic patterns in the supraclavicular and cervical lymph nodes.

Although complete comparison is difficult due to the different radiation dosages from the chest radiograph at the 2 hospitals, pneumonic infiltration at Hospital “A” was shown to be decreased at Hospital “B”. It appeared that pneumonitis developed from complicated pneumoconiosis when visiting Hospital “A”, and improved afterwards. While there were no unusual finding in bronchoscopy at Hospital “A”, it could be seen in bronchoscopy at Hospital “B” that fibrosing stenosis has progressed as segmental bronchi within right middle, right upper, and left upper lobes have become narrower.

Bronchial anthracosis may cause bronchial pigment discoloration and fracture deformation, and various studies exist, reporting both the relevance and irrelevance of anthracosis with TB [16-18]. Also, debates on the relevance of anthracosis and smoking are ongoing [19,20]. It is observed in the bronchoscopy and the CT that anthracosis occurring in the lungs is accompanied by bronchial stenosis, atelectasis, pigmentation of the lung, hypertrophy of the lymph nodes, and calcification of the lymph nodes [20].

Since the occurrence of extrathoracic anthracosis is rare, its pathogenesis and prognosis are not clearly defined, and there have been some case reports of its occurrences in the spleen, liver, esophagus, sinuses, and retroperitoneum in Korea and other countries [21-26]. A few cases that required distinguishing anthracosis from other tumorous diseases exist, including the one [27] showing a pseudotumor in the mediastinal lymph nodes and the other [26] of retroperitoneal silicosis mass that was suspected as pancreatic cancer in a miner suffering from silicosis for 30 years. Although the extrathoracic route of anthracosis is unclear, it is likely that it moves via lymphohematogenous spread [28-30], and we concluded that it should be determined whether the case in this report also follows such a path by examining a number of cases in the future. In addition, whether cervical anthracosis means exposure to a variety of dusts needs to be studied.

## Consent

Written informed consent was obtained from the patient for the publication of this report and any accompanying images.

## Competing interests

The authors declare that they have no competing interests.

## Authors’ contributions

HSY and JIK interviewed the patient and wrote the article. TJY and SWL identified relevant references. SWL performed the estimation for the environmental assessment. BJY and KYJ provided expertise in clinical medicine. All of the authors read and approved the final manuscript.
